# Modelling precise responses to anti‐seizure medication using brain organoids carrying *SCN2A*‐GoF and *SCN2A*‐LoF mutations

**DOI:** 10.1002/ctm2.70666

**Published:** 2026-04-21

**Authors:** Yuling Yang, Yi Yan, Yang Cai, Xin Wang, Zhicheng Shao, Jing Ding

**Affiliations:** ^1^ Department of Neurology Zhongshan Hospital, Fudan University Shanghai China; ^2^ State Key Laboratory of Medical Neurobiology and MOE Frontiers Center for Brain Science, Fudan University Shanghai China; ^3^ Institute for Translational Brain Research, Fudan University Shanghai China

1

Dear Editor,

Epilepsy is characterised by excessive and synchronised neuronal discharges, yet its treatment is complicated by high inter‐individual variability in drug responses. As a paradigmatic example, patients with *SCN2A* mutations exhibit remarkably different clinical outcomes: those with gain‐of‐function (GoF) variants often benefit from sodium channel blockers (SCBs), whereas those with loss‐of‐function (LoF) variants may experience seizure exacerbation upon SCB administration.^1^ To address the need for mutation‐specific therapy strategies, we generated human induced pluripotent stem cell (iPSC)‐derived cortical organoids carrying *SCN2A*‐GoF and *SCN2A*‐LoF variants. Our study demonstrates that these brain organoids faithfully recapitulate genotype‐specific pharmacological responses, offering a promising preclinical platform for precision medicine in *SCN2A*‐related epilepsy.

In our earlier research, we discovered and described a new *SCN2A* mutation (E512K) in an epilepsy patient, which showed a GoF impact on sodium channel function.^2^ Recently, the patient presented with recurrent seizures, and adjustment of anti‐seizure medication to carbamazepine (CBZ) resulted in no further clinical epileptiform activity. On the other hand, we identified a different novel *SCN2A* mutation (N916S) in a family where twin brothers presented with febrile seizures (FS) and generalised epilepsy with FS plus (GEFS+) (Figure 1A). Video‐EEG revealed generalised spike‐wave, polyspike‐wave and sharp‐wave patterns in the index patient and his identical twin (Figure 1C,D). The parents were asymptomatic, although the mother had an EEG with generalised spike‐wave activity. Treatment of the proband with oxcarbazepine (OXC) exacerbated seizures, whereas levetiracetam (LEV) demonstrated good efficacy. We performed whole exome sequencing (WES) on the index patient and Sanger sequencing for family verification. We detected a heterozygous *SCN2A* mutation (c.2747A > G, Figure 1E) in the index patient, his twin and their mother (Figure 1A,B). To evaluate how this new mutation affects channel electrophysiology and to explore genotype‒phenotype correlations, we measured sodium currents in HEK293T cells expressing wild‐type (WT) or mutant Nav1.2 channels in both adult and neonatal isoforms. As shown in Figures 1 and S1, the N916S variant caused a LoF effect in both isoforms, with more pronounced deficits in the adult isoform, including a notable hyperpolarising voltage shift in steady‐state inactivation and prolonged recovery time from fast inactivation (Figure 1L–Q). These observations from patients reinforce that SCBs are effective for *SCN2A*‐GoF mutations but can aggravate seizures in cases with *SCN2A*‐LoF mutations.

**FIGURE 1:**
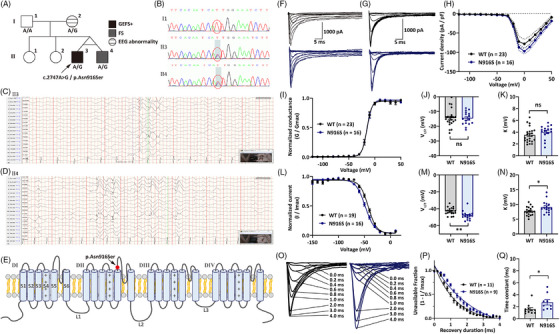
Clinical features and functional assessment of a novel *SCN2A* variant in identical twin siblings. (A) Pedigree chart illustrating the index case and family relatives. Male symbols are boxes, female ones circles. Pointer marks the index case. (B) Sanger sequencing results for the *SCN2A* gene. The index case carried a heterozygous missense change c.2747A > G (p.N916S). This change appeared in his mother and identical twin but was absent in his father. (C and D) EEG patterns of the index case and his twin sibling. (E) Diagram showing the Nav1.2 protein structure with the position of the N916S variant. (F) Representative sets of sodium currents during activation in the adult isoforms. (G) Representative sets of sodium currents during inactivation in the adult isoforms. (H) Curves depicting peak current density against voltage. Currents were adjusted for cell capacitance. (I) Graphs of normalised conductance fitted to Boltzmann against voltage. (J) Bar graph of the voltage where half the sodium channels activate. (K) Bar graph of the activation slope factor (K). (L) Graphs of normalised current (*I*/*I*_max_) against voltage for inactivation, fitted to Boltzmann. (M) Bar graph of the voltage where half the sodium channels inactivate. (N) Bar graphs of (K) for inactivation. (O) Illustration of recovery currents after varying intervals following a 50‐ms inactivation pulse at ‒10 mV in the adult isoforms. (P) Recovery from inactivation over time. Curves represent single exponential fittings. (Q) Bar graph of the recovery time constant from inactivation. Bars show mean ± SEM. Differences between wild‐type (WT) and N916S were evaluated with unpaired two‐tailed *t*‐test. ^*^ *p* < .05, ^**^ *p* < .01.

Given that the efficacy of SCBs in *SCN2A*‐related epilepsy is highly dependent on the underlying mutation, we sought to validate brain organoids as a predictive model. This approach allowed us to establish a reliable preclinical platform to screen for drug responses that align with patient‐specific outcomes. Due to the inability to obtain iPSCs from patients in the second family, we utilised iPSCs derived from the patient with *SCN2A*‐E512K mutation as we previously reported.^2^ Using CRISPR‐Cas9 based gene editing, we generated an isogenic control by correcting the mutation, as well as an additional cell line carrying an indel mutation (classified as LoF). As shown in Figure 2A, this indel resulted in a frameshift truncation mutation (p.Lys511Argfs*5) caused by the deletion of 17‐bp nucleotides (c.1530_1546del). Patch‐clamp recordings confirmed that this truncation variant produced no detectable sodium currents in transfected cells, validating it as a reliable LoF allele for modelling *SCN2A* haploinsufficiency (Figure S2). Subsequently, we induced cortical organoids from these three iPSCs, yielding organoids carrying heterozygous *SCN2A*‐GoF, *SCN2A*‐WT and *SCN2A*‐LoF mutations with same genetic background. Then, electrophysiological recordings were performed using multi‐electrode arrays (MEAs) (Figure 2A). As illustrated in Figure 2B–G, MEA recordings revealed that both *SCN2A*‐GoF and *SCN2A*‐LoF mutations led to increased spiking, bursts and network synchronisation in cortical organoids compared to isogenic (*SCN2A*‐WT) controls. To assess the pharmacological efficacy of CBZ in our model, we first evaluated the response of isogenic WT organoids to CBZ. MEA recordings demonstrated that 30 µM CBZ significantly suppressed spontaneous neuronal activity in WT organoids (Figures 2B,E–G). This confirms the expected inhibitory action of CBZ as a SCB in a normal genetic background. However, in the context of mutation‐induced hyperexcitability, CBZ exhibited vastly different effects depending on the functional type. In *SCN2A*‐GoF organoids, CBZ significantly suppressed neuronal hyperactivity, reducing spiking frequency, burst frequency and synchrony index (Figure 2C,E–G). In contrast, CBZ failed to suppress hyperactivity in LoF organoids (Figures 2D,E–G). Instead, CBZ administration even resulted in a paradoxical effect, where some LoF organoids were induced to break out network bursts (Figure 2H–K). The emergence of network bursts indicated enhanced synaptic transmission in *SCN2A*‐LoF organoids after the application of CBZ.

**FIGURE 2:**
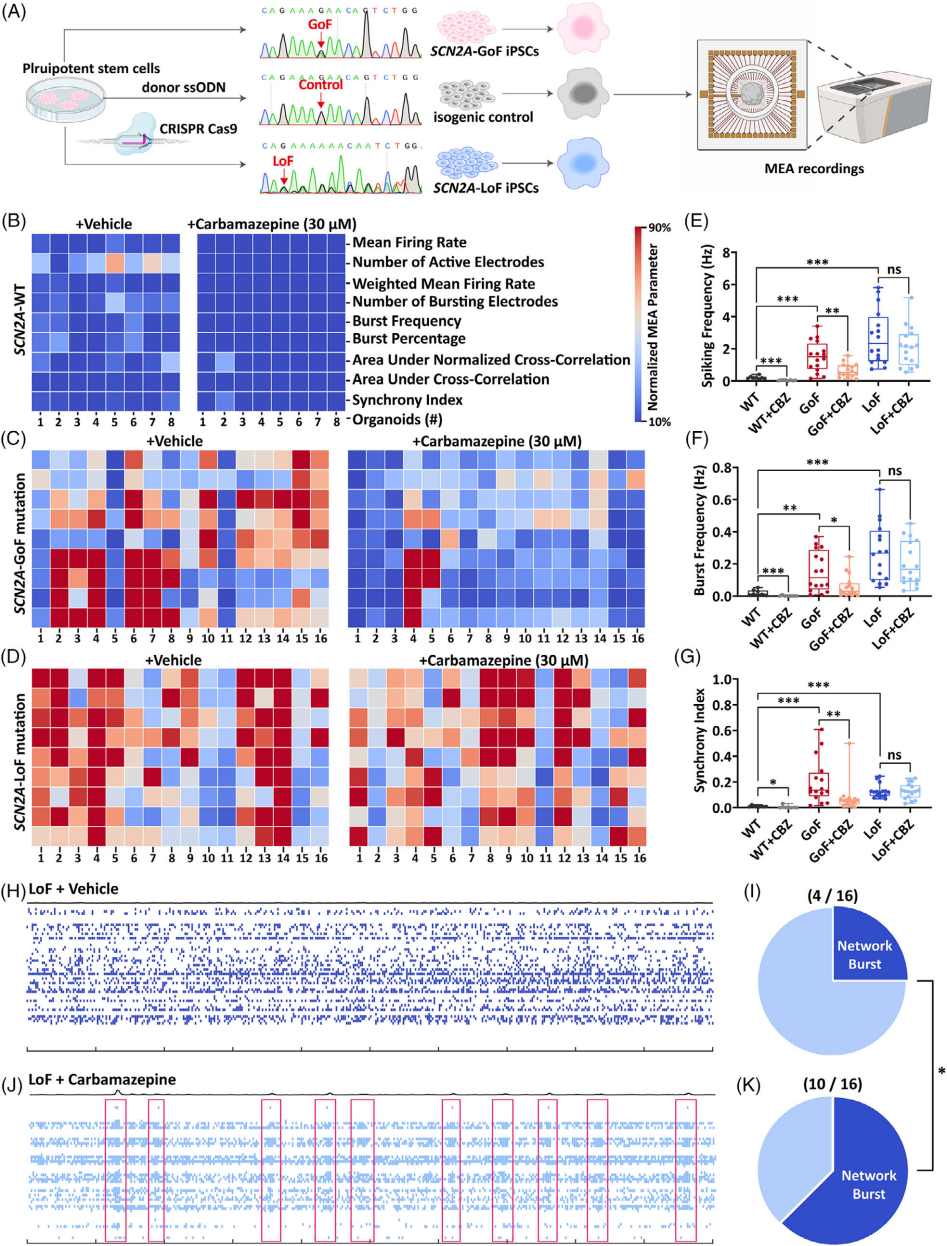
Cortical organoids with *SCN2A*‐gain‐of‐function (GoF) and *SCN2A*‐loss‐of‐function (LoF) mutations showed hyperexcitability but opposite responses to carbamazepine. (A) Overview of the process for induced pluripotent stem cell (iPSC) creation, organoid development and multi‐electrode array (MEA) assessments. (B) Heatmap displaying main MEA parameters in *SCN2A*‐wild‐type (WT) organoids under vehicle or 30 µM carbamazepine. *n* = 8 organoids. (C and D) Heatmap displaying main MEA parameters in *SCN2A*‐GoF (C) and *SCN2A*‐LoF (D) organoids following 5‐min exposure to vehicle or 30 µM carbamazepine. *n* = 16 organoids. (E–G) Measures of spike rate, burst rate and synchrony index from *SCN2A*‐WT, *SCN2A*‐GoF and *SCN2A*‐LoF organoids before and after 30 µM carbamazepine. (H–K) Sample raster plots and pie charts indicating the fraction of organoids showing network bursts in *SCN2A*‐LoF organoids prior to and following 30 µM carbamazepine. Boxplots: median at centre; 25% quantile at lower hinge; 75% quantile at upper hinge. Differences between groups were assessed with Mann–Whitney test (E–G) and chi‐square test (I and K). ^*^ *p* < .05, ^**^ *p* < .01, ^***^ *p* < .001.

The increase in network bursts is associated with two main factors, including the enhanced neuronal excitability and the strengthening of synaptic transmission.^3^, ^4^ CBZ can reduce neuronal excitability by maintaining the inactivated state of sodium channels, but its effects on synaptic transmission are controversial. Actually, CBZ has been reported to promote the excitatory synaptic current.^5^ Therefore, to explore the mechanism underlying the opposite effects of CBZ on GoF and LoF cortical organoids, we performed bulk RNA sequencing. Notably, although the expression levels of sodium ion channels showed reduced functionality, synapse‐associated genes were considerably elevated in the LoF group (Figure 3A–C). The results suggested that both presynaptic and postsynaptic elements were increased in *SCN2A*‐LoF organoids (Figure 3D,E), implying that the excessive excitation could be driven by augmented synaptic activity. Furthermore, CBZ might have a facilitating effect on excitatory synaptic transmission,^5^ ultimately leading to an abnormal increase in network bursts.

**FIGURE 3:**
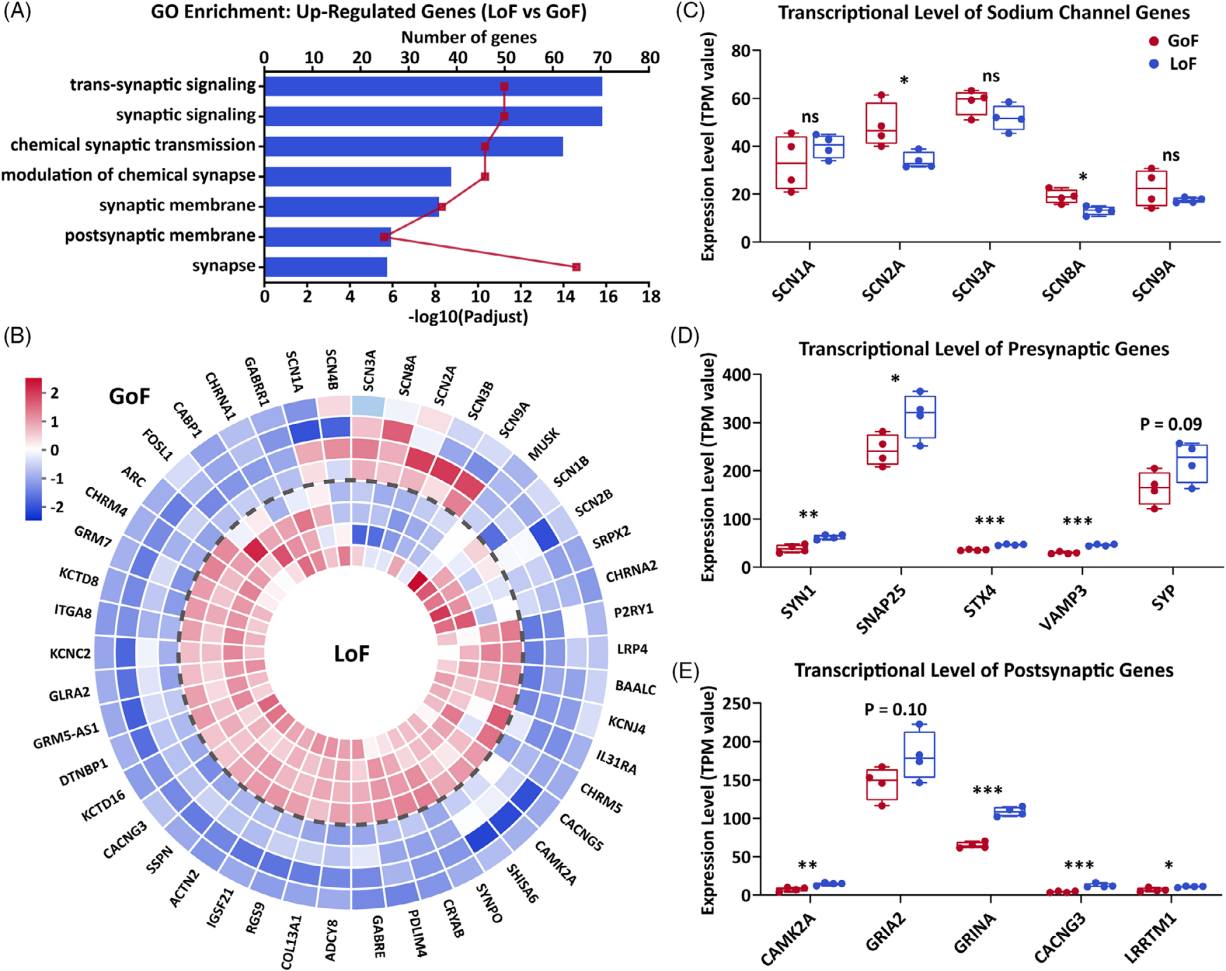
Transcriptional profiling revealed upregulation of genes associated with synapses in *SCN2A*‐loss‐of‐function (LoF) organoids. (A) Gene Ontology (GO) term enrichment of upregulated genes in *SCN2A*‐LoF organoids. *n* = 4 organoids. (B) Heatmap showing the relative expression of genes associated with voltage‐gated sodium channels and synapses in *SCN2A*‐gain‐of‐function (GoF) and *SCN2A*‐LoF organoids. (C–E) Boxplots showing the Transcripts Per Million (TPM) value of representative genes in *SCN2A*‐GoF and *SCN2A*‐LoF organoids. Boxplots: median at centre; 25% quantile at lower hinge; 75% quantile at upper hinge. Differences between *SCN2A*‐GoF and *SCN2A*‐LoF were evaluated with unpaired two‐tailed *t*‐test (C–E). ^*^ *p* < .05, ^**^ *p* < .01, ^***^ *p* < .001.

To summarise, our study underscores the importance of patient‐specific therapies and highlights the application of brain organoid modelling for precision approaches in treating epilepsy. However, it is important to acknowledge that *SCN2A*‐LoF encompasses a spectrum of pathogenic mechanisms, including altered gating kinetics and protein instability. Our 17‐bp deletion strategy specifically represents *SCN2A* haploinsufficiency, which may differ from missense LoF variants that retain some non‐functional protein expression. Therefore, future research incorporating a wider array of LoF variants is necessary to fully map the genotype–phenotype correlations in *SCN2A*‐related epilepsy.

## AUTHOR CONTRIBUTIONS

Yuling Yang and Yi Yan conducted most of the experiments and analysed the data, including gene editing, organoid culture and multi‐electrode array recordings. Yang Cai worked on collecting clinical information and EEG recordings. Yuling Yang prepared the original draft. Jing Ding, Zhicheng Shao and Xin Wang designed the study and reviewed the paper.

## CONFLICT OF INTEREST STATEMENT

None of the authors has any conflict of interest to disclose.

## FUNDING INFORMATION

This study was supported by funding from the National Key Research and Development Program of China (code: 2022YFC2503802).

## ETHICS STATEMENT

This study was approved by the ethical committees at the Zhongshan Hospital of Fudan University (approval code: B2022‐431R; approval date: 13 September 2022).

## Supporting information



Supporting Information

## Data Availability

The data that support this study are available from the corresponding author upon reasonable request.
