# Supplementary material for: Modelling precise responses to anti‐seizure medication using brain organoids carrying SCN2A‐GoF and SCN2A‐LoF mutations
Source: Clin Transl Med. 2026 Apr 21;16(4):e70666. doi: 10.1002/ctm2.70666 (PMC13097342; doi:10.1002/ctm2.70666)
Supplement: Supplementary file 1 — Supporting Information [file CTM2-16-e70666-s001.docx]

**——Supplementary Materials——**

**Modeling Precise Responses to Anti-Seizure Medication Using Brain Organoids Carrying *SCN2A*-GoF and -LoF Mutations**

Yuling Yang^1^, YiYan^1^, Yang Cai^1^, Xin Wang ^1^, Zhicheng Shao^2,3 *^, Jing Ding^1,2 *^

**Author affiliation:**

^1^Department of Neurology, Zhongshan Hospital, Fudan University, Shanghai, 200032, China

^2^State Key Laboratory of Medical Neurobiology and MOE Frontiers Center for Brain Science, Fudan University, Shanghai, 200032, China

^3^Institute for Translational Brain Research, Fudan University, Shanghai 200032, China

**Methods**

**Ethics approval and consent to participate**

This study was conducted in compliance with the ethical standards of the Zhongshan Hospital to Fudan University. Informed consent was obtained from patients and their relative family members.

**Genetic analyses**

Whole-exome sequencing (WES) was performed in the twin brothers and their parents to identify the causative gene. Peripheral blood samples were collected from the patients and their relative families. Sanger sequencing was performed to validate the putative pathogenic variants.

**Mutagenesis**

The wild-type recombinant human *SCN2A* plasmid of adult isoform (pcDNA3.1-*SCN2A*-3xFLAG) was purchased from MiaoLingPlasmid (Wuhan, China). 3xFLAG was deleted by targeted deletion mutation and validated by full-length sanger sequencing to acquire pcDNA3.1-*SCN2A*. To generate the neonatal isoform, a single substitution (D209N) was introduced. The N916S variant was introduced into both 5N and 5A isoforms. The 17-bp deletion mutation (c.1530_1546del) was introduced into the adult isoform using site-directed mutagenesis. All the constructed plasmids were verified by resequencing before transfection into HEK293T cells. All primers are available upon request.

**Cell culture and transfection**

Heterologous expression of WT and mutated plasmids was performed in Human embryonic kidney 293T (HEK293T) cells. HEK293T cells were seeded in 24-well plates and cultured in humidified air and 5% CO_2_ at 37 °C. The medium for culture was Dulbecco’s modified Eagle’s medium (Gibco, USA) supplemented with 10% fetal bovine serum (Gibco, USA), and 1% penicillin/streptomycin (Gibco, USA). Plasmids expressing either wild-type or mutated *SCN2A* (2 μg) were transiently transfected together with EGFP (1 μg) into HEK293T cells using Lipofectamine 2000 (Thermo Fisher Scientific, Waltham, MA, USA) when cells were approximately 90% confluent.

**Electrophysiological recordings**

Electrophysiological recordings from fluorescent cells were made 36-48h after transfection at 25 °C using Multiclamp 200B amplifier (Axon, USA), Digidata 1440A digitizer (Axon, USA), and pCLAMP 10.3 software (Axon, USA). To record sodium current mediated by Nav1.2, the recording pipettes were pulled from borosilicate glass by pipette puller PC-100 (Narishige, JPN), with the resistance of 3-5 MΩ when ﬁlled with intracellular recording solution containing (in mM): 110 CsF, 10 NaCl, 20 EGTA and 10 HEPES. pH was adjusted to 7.2 with 0.5 M CsOH and the osmolarity was adjusted at ∼290 mOsm. The bath solution was consisted of (in mM) 135 NaCl, 4.5 KCl, 1 MgCl2, 2 CaCl2, 5 D-glucose, 10 HEPES (pH 7.4 with NaOH, ∼300 mOsm). Standard whole-cell currents were filtered at 2 kHz, recorded at 10 or 100 kHz. The pipette potential was adjusted to zero before seal formation. Capacity transients were canceled, and the voltage errors were minimized with 80% series resistance compensation.

To assess the current-voltage (I–V) relationship, cells were held at −120 mV and currents were elicited by 100-ms test pulses from −120 to +50 mV at 10-mV increments at 5-s intervals. The peak sodium currents were measured, normalized with cell capacitance, and plotted against test voltage to generate the I–V plot. The activation curves were fitted by the Boltzmann function: G/Gmax = 1/ (1 + exp [(V − V_1/2_)/k] with V_1/2_ being the voltage of half-maximal activation and k a slope factor. For steady-state fast inactivation, cells were held at −120 mV and currents were determined by a series of 500-ms prepulses from −150 to 0 mV in 10-mV increments followed by a second pulse to −10 mV for 50 ms to assess channel availability. The peak currents at the test pulse were normalized to the maximum current and the normalized current was plotted against the prepulse voltage to obtain the inactivation curve. The inactivation curve was then fitted to the Boltzmann function as follows: I/Imax = 1/ (1 + exp [(V − V_1/2_)/k]. For recovery from inactivation, cells were held at − 120 mV and depolarized to a test potential of −10 mV for 50-ms to inactivate Na+ channels and then repolarized to − 120 mV for increasing duration (0-ms to 4-ms at 0.2-ms increments) followed by a 50-ms pulse to −10 mV to assess the extent of channel recovery. Peak currents were normalized by maximum currents and fitted to the one-phase exponential decay function: I/Imax = A [exp (− t/τ_rec_)] to determine the time constant τ.

**hiPSC cell culture**

The iPSC cells were grown on 1% Matrigel (Corning, #354230) coated plate in Nutristem medium (Biological Industries, #05-100-1A) in 5% CO2 incubator at 37 ℃ and passaged when colonies reached about 85% confluency using TrypLE (Gibco, #12604021). Y-27632 (10 μM, Selleck, #129830-38-2) was added for the initial 12 h after passage.

**CRISPR–Cas9 based genome editing**

The iPSCs with an *SCN2A* LoF mutation were generated in a cell line derived from a patient harbouring *SCN2A*-GoF mutation with CRISPR/Cas9 as we previously reported^4^. Briefly, when iPSCs growed to 85% confluency, dissocation and nucleofection were performed. 800 thousand cells were nucleofected (Lonza Amaxa 4D nucleofector, program CB-150) with 12 μg NLS-Cas9-NLS protein (GenCrispr, #Z03469-100) and 200 pmol single guide RNA: 5′-AAGAAACAGAAAAAACAGTC-3′(GenCrispr). The cells were seeded onto one well of a Matrigel coated 6-well plate in Nutristem medium. When colonies reached about 85% confluency, single cell was seeded into 96-well plates. Following the cloning of single-cell derived colonies, genomic DNA was isolated by rapid DNA Extraction kit (Beyotime, #D0065S). The target region was amplified by PCR and subjected to Sanger sequencing.

**Cortical organoids generation and culture**

The induction protocol for cortical organoids can be referenced from our previous work^4^. A key difference was that we cultured the organoids using BrainPhys Neuronal Medium and SM1 (Stemcell Technologies, # 05792) containing 10 ng/ml BDNF (Peprotech, #AF-450-02), 10 ng/ml GDNF (Peprotech, #AF-450-10) and 200 μM L-Ascorbic Acid (Sigma, #A8960) from day 21 on, to promote the electrical maturation of cortical organoids.

**MEA recordings and drug application**

Organoids cultured during 80-90 day were plated on CytoView MEA 6 (Axion Biosystems, USA) and the electrical activities of organoids were recorded using Maestro Edge (Axion Biosystems). Before the application of Carbamazepine (CBZ, sigma, #94496), organoids were first recorded for 5 min in the culture medium containing vehicle (DMSO) to obtain the baseline measurement. Then, organoids were incubated in the culture medium containing 30 μM CBZ for 10-min equilibrium. Afterward, another 5-min recording was made to test the CBZ effects.

**Bulk RNA sequencing of organoids and analysis**

Total RNA of each organoid was extracted using RNAiso Plus (Takara, #9109) according to the manufacturer’s instructions. Only high-quality RNA sample (OD260/280 =1.8~2.2, OD260/230 ≥ 2.0, RQN ≥ 7.0, 28S:18S ≥ 1.0) was used to construct sequencing library. RNA purification, reverse transcription, library construction and sequencing were performed at Shanghai Majorbio Bio-pharm Biotechnology Co., Ltd. (Shanghai, China). Differential expression analysis was performed using the DESeq. DEGs with |log_2_FC| > 1 and pvalue < 0.05 (DESeq2) were considered to be significantly different expressed genes.

**Statistical analysis**

All data were presented as Boxplots. Statistical analyses and graphic representations were performed using Prism GraphPad 8.0. The Mann-Whitney U test, unpaired two-tailed t-test, or Chi-Square test was used to assess the statistical significance of differences in two groups. When *P* < 0.05, differences were accepted as significant. **P* < 0.05, ***P* < 0.01, ****P* < 0.001.

**Supplementary Figures and Figure Legends**


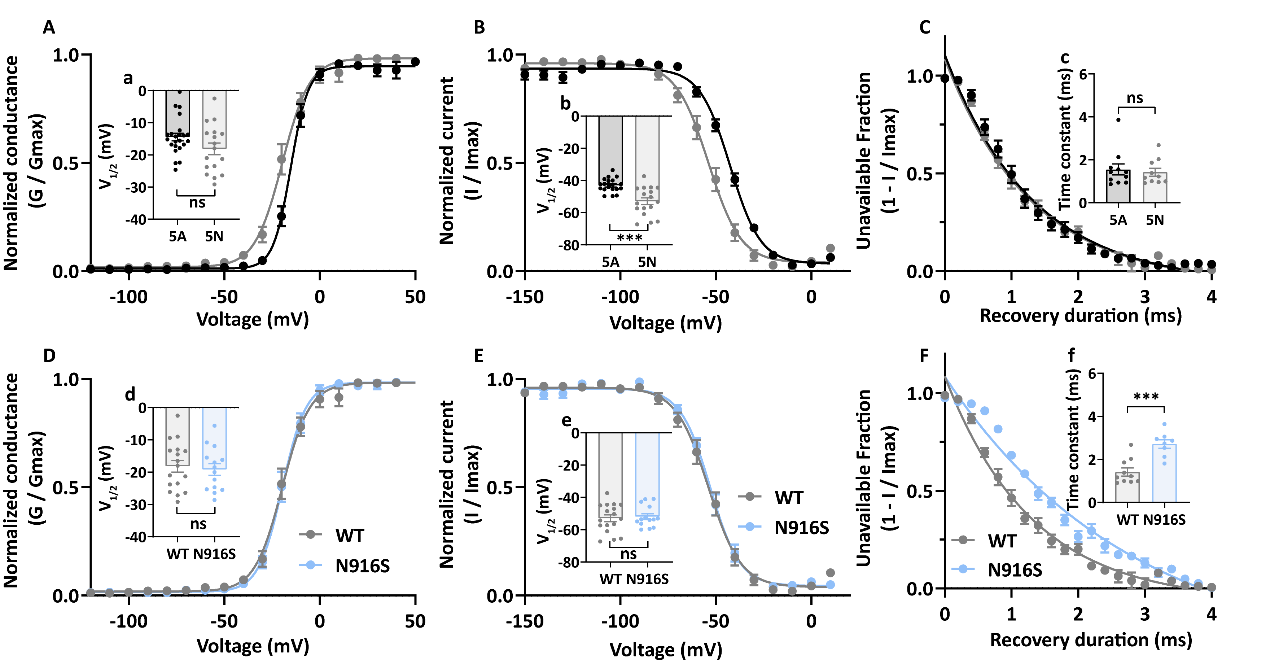


**Supplementary Figure 1.** **Functional Properties of WT and Mutant Neonatal Nav1.2 Channels.** (A) Plots of Boltzmann fits of normalized conductance versus membrane potential when determining steady-state activation for adult and neonatal WT Nav1.2 channels and histogram of V_1/2_. (B) Plots of normalized current versus membrane potential of steady-state fast inactivation for adult and neonatal WT Nav1.2 channels and histogram of V_1/2_. (C) Time dependence curves of recovery from fast inactivation and histogram of the time constant for adult and neonatal WT Nav1.2 channels. (D) Plots of Boltzmann fits of normalized conductance versus membrane potential for 5N-WT and 5N-N916S and histogram of V_1/2_. (E) Plots of normalized current of steady-state fast inactivation for 5N-WT and 5N-N916S and histogram of V_1/2_. (F) Single exponential fits of the recovery data for 5N-WT and 5N-N916S, and histogram of the time constant of recovery from inactivation. Differences were evaluated via an unpaired two-tailed t-test. ****P* < 0.001.


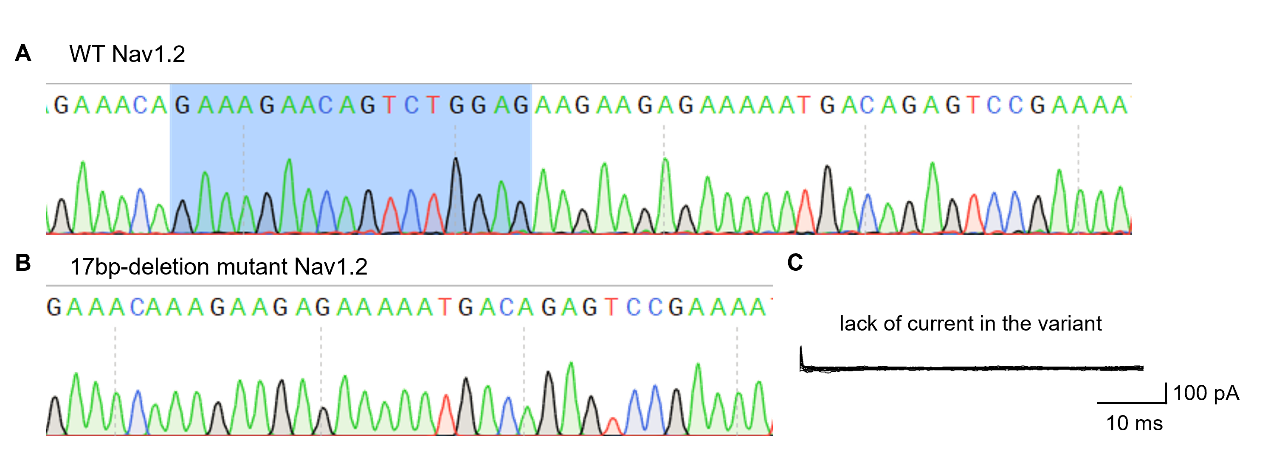


**Supplementary Figure 2.** **Validation of the *SCN2A* 17-bp deletion mutant.** (A) Sanger sequencing of the WT plasmid. (B) Sanger sequencing of the mutant plasmid. The highlighted region confirms the 17-bp deletion (c.1530_1546del). (C) Representative traces from HEK293T cells transiently expressing the 17-bp deletion mutant.

**Supplementary Table 1.** **Biophysical properties of N916S variant in adult and neonatal isoforms.**

| Variant | isoform | V_1/2_ activation  (mV) | K activation  (mV) | V_1/2_ inactivation  (mV) | K inactivation  (mV) | Tau fast  (ms) | Tau slow  (ms) | Persistent current  (%) | Recovery time  (ms) |
| --- | --- | --- | --- | --- | --- | --- | --- | --- | --- |
| WT | Adult | -14.5 ± 1.2 | 3.5 ± 0.3 | -43.0 ± 1.0 | 7.7 ± 0.3 | 2.3 ± 0.3 | 6.9 ± 0.5 | 11.6 ± 1.0 | 1.5 ± 0.3 |
| WT | Neonatal | -18.1 ± 1.8 | 5.9 ± 0.7 | -53.0 ± 2.1 | 7.1 ± 0.6 | 0.9 ± 0.1 | 3.6 ± 0.6 | 8.8 ± 1.3 | 1.4 ± 0.2 |
| N916S | Adult | -14.7 ± 1.1 | 3.5 ± 0.3 | -48.2 ± 1.3** | 9.1 ± 0.5* | 2.8 ± 0.4 | 8.6 ± 1.0 | 9.7 ± 1.2 | 2.7 ± 0.4* |
| N916S | Neonatal | -19.2 ± 1.8 | 4.7 ± 0.5 | -52.0 ± 1.8 | 6.5 ± 0.4 | 1.1 ± 0.2 | 3.8 ± 0.7 | 8.0 ± 1.6 | 2.7 ± 0.2*** |

**P* < 0.05, ***P*< 0.01, and ****P* < 0.001 vs WT of the same isoform.
